# Genome-wide analytical approaches for reverse metabolic engineering of industrially relevant phenotypes in yeast

**DOI:** 10.1111/j.1567-1364.2011.00776.x

**Published:** 2012-01-10

**Authors:** Bart Oud, Antonius J A Maris, Jean-Marc Daran, Jack T Pronk

**Affiliations:** Department of Biotechnology, Delft University of Technology and Kluyver Centre for Genomics of Industrial FermentationDelft, The Netherlands

**Keywords:** inverse metabolic engineering, whole genome sequencing, transcriptomics, systems biology, yeast, evolutionary engineering

## Abstract

Successful reverse engineering of mutants that have been obtained by nontargeted strain improvement has long presented a major challenge in yeast biotechnology. This paper reviews the use of genome-wide approaches for analysis of *Saccharomyces cerevisiae* strains originating from evolutionary engineering or random mutagenesis. On the basis of an evaluation of the strengths and weaknesses of different methods, we conclude that for the initial identification of relevant genetic changes, whole genome sequencing is superior to other analytical techniques, such as transcriptome, metabolome, proteome, or array-based genome analysis. Key advantages of this technique over gene expression analysis include the independency of genome sequences on experimental context and the possibility to directly and precisely reproduce the identified changes in naive strains. The predictive value of genome-wide analysis of strains with industrially relevant characteristics can be further improved by classical genetics or simultaneous analysis of strains derived from parallel, independent strain improvement lineages.

## Introduction

Metabolic engineering, the targeted and knowledge-based modification of cellular processes by genetic modification with the aim to improve industrial performance (Bailey *et al*., 1990; Nielsen, 2001), is a key driver for progress in yeast biotechnology. Novel yeast-based processes for production of a wide range of chemical compounds, ranging from pharmaceuticals to bulk chemicals and biofuels, are intensively investigated and increasingly find their way toward industrial implementation (Nevoigt, 2008; Abbott *et al*., 2009; Tsuruta *et al*., 2009; Sauer *et al*., 2010; Zhang *et al*., 2011). Fifteen years after the first complete *Saccharomyces cerevisiae* genome sequence became available (Goffeau *et al*., 1996), functional genome analysis, quantitative physiology, and systems biology have advanced our understanding of yeast metabolic networks to such an extent that knowledge-based genetic intervention increasingly yields the intended positive impacts on industrial performance. Current developments in automated, high-throughput strain construction and analysis (Wang *et al*., 2009a; Anderson *et al*., 2010) and synthetic biology techniques for rapid synthesis and manipulation of DNA sequences (Gibson, 2011) further accelerate progress in knowledge-based metabolic engineering.

Despite the growing number of successes in yeast metabolic engineering, many cases remain in which the current level of understanding is insufficient to achieve the quantum leaps in performance demanded by industry. Knowledge-based engineering of traits such as pathway kinetics, cellular energetics, and robustness represent not only relevant and intellectually stimulating, but also painstaking and time-consuming challenges. Examples of such challenges include the long-running attempts to engineer industrially relevant aspects of yeast physiology such as glycolytic flux (Kern *et al*., 2007) and tolerance to ethanol and acetic acid (Mira *et al*., 2010; Stanley *et al*.,2010a, b). Consequently, there is a growing awareness in academia and industry that fast improvement of microbial strains requires integration of targeted metabolic engineering with modifications that do not *a priori* target specific genes (from here on referred to as nontargeted approaches) (Lee *et al*., 2011).

Nontargeted approaches for strain improvement have been a key driver in microbial biotechnology for over half a century. Even in the absence of detailed knowledge of genetics or physiology of the producing strain, their effectiveness is beyond dispute. The paradigm of such ‘classical’ strain development is the huge improvement, over a period of 60 years, of penicillin production by the filamentous fungus *Penicillium chrysogenum* (Nielsen, 1997; van den Berg *et al*., 2008). However, nontargeted strain improvement typically leads to a slow, incremental increase in performance, especially in the later stages of strain improvement. Moreover, its ‘black box’ character precludes the rapid transfer of relevant traits among strains or species. To address these limitations, it is essential to identify the genetic changes and mechanisms that underlie the improved performance of strains generated via nontargeted approaches.

In many technological disciplines, ranging from military to medical engineering, the process of elucidating the technological principles of a system via analysis and subsequent reconstruction of its structure and function is known as reverse engineering (Sutton, 1984; Rekoff, 1985). In a seminal paper by Bailey and co-workers (Bailey *et al*., 1996), this concept was introduced to the field of biotechnology as ‘inverse’ metabolic engineering. However, to maintain consistency with other engineering disciplines, ‘reverse’ metabolic engineering is used throughout this mini-review.

**Fig. 1:**
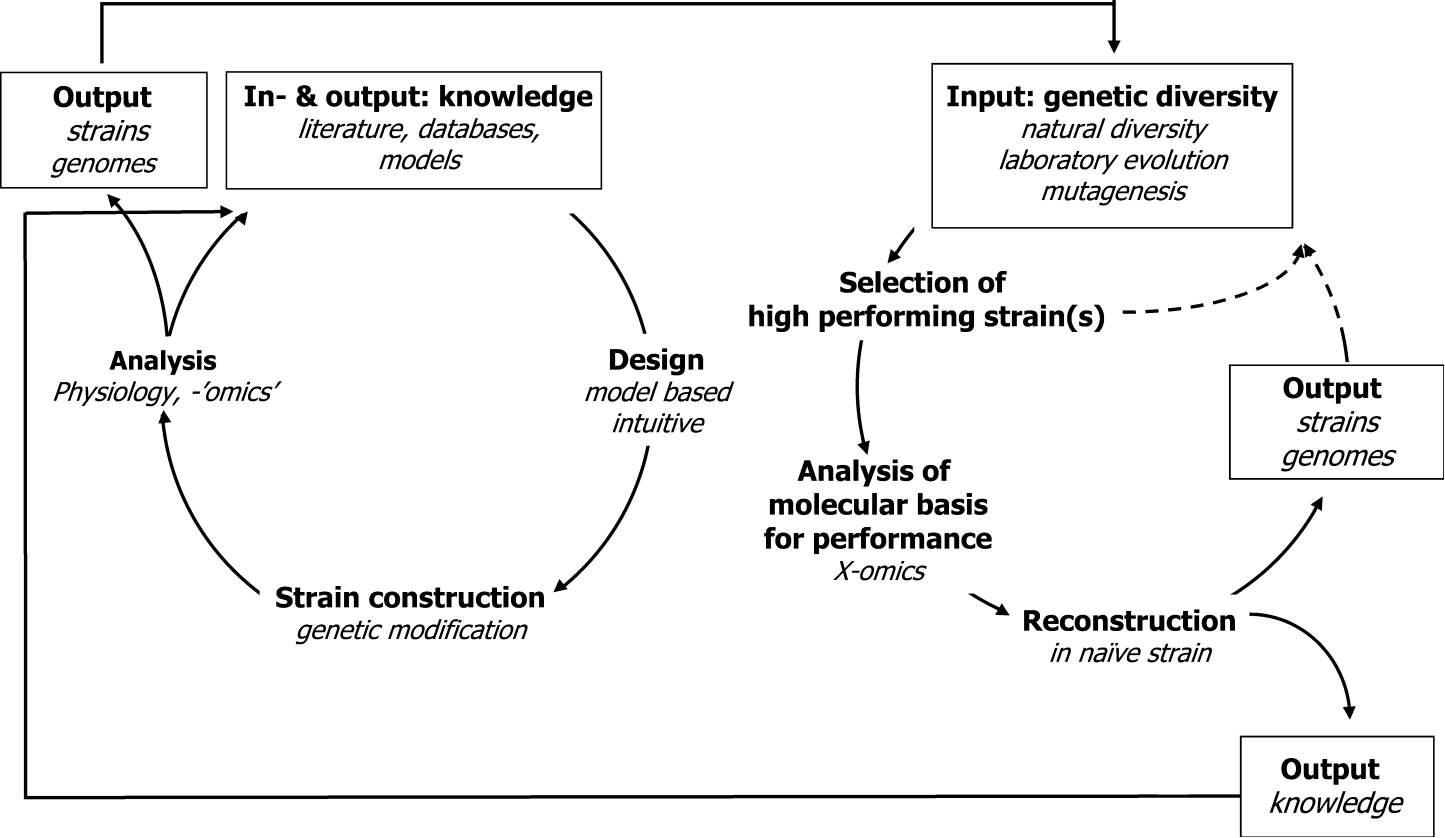
The ‘forward’ metabolic engineering and ‘reverse’ metabolic engineering cycles and their interaction. In forward metabolic engineering, analysis of strains constructed based on rational design often results in scientific questions that need to be addressed by further analysis and consultation of the rapidly expanding knowledge on microbial metabolism and its regulation. In reverse metabolic engineering, generation and analysis of biodiversity – obviously, with special attention for strains that show improved performance – contributes to accelerated strain improvement and knowledge development (After Nielsen, 2001; Bailey *et al*., 1996; Bro & Nielsen, 2004).

In contrast to the conventional ‘forward’ metabolic engineering cycle, which starts with a knowledge-based design that is subsequently tested by construction and analysis (Fig. 1), reverse metabolic engineering starts out with (an) existing microbial strain(s) with improved performance relative to (a) reference strain(s). High-performing strains can be either isolated from nature, obtained from culture collections, or created through nontargeted strain improvement efforts. Such nontargeted approaches for optimization of yeast strains include the following: (i) random mutagenesis combined with high-throughput selection, such as UV-C mutagenesis of the xylose-fermenting yeast *Scheffersomyces stipitis* for improved fermentation characteristics under anaerobic conditions (Hughes *et al*., 2011); (ii) laboratory evolution (‘evolutionary engineering’) under cultivation regimes that have been especially designed to convey a selective advantage to better performing strains (reviewed by Conrad *et al*., 2011). An example of this approach is the improvement of the fermentation kinetics of genetically engineered *S. cerevisiae* strains during growth on glucose-xylose-arabinose mixtures via evolutionary engineering (Wisselink *et al*., 2009); and (iii) the introduction of gene libraries, sometimes after mutagenizing the expressed genes. This has for instance been applied to transcription factor engineering of *S. cerevisiae* for improved ethanol tolerance (Alper *et al*., 2006).

The next step of the reverse metabolic engineering cycle is the elucidation of the genetic basis for improved performance (Fig. 1). This elucidation should be rigorous and move beyond merely establishing genotype–phenotype correlations. Instead, it should be unambiguously demonstrated that reintroduction of a defined set of genetic changes can wholly or partially reconstruct the improved performance. Reverse metabolic engineering has the added benefit that it enables the extraction of productive mutations, thereby avoiding the accumulation of nonproductive mutations that may occur in prolonged nontargeted strain improvement programmes.

Integration of the ‘forward’ and reverse metabolic engineering cycles can be accomplished in several ways. Once the molecular basis for improved performance, preferably including understanding of the underlying biochemical mechanism, has been elucidated by a reverse engineering approach, this knowledge can be implemented in ‘forward’ metabolic engineering of the same strain lineage or of other strains (Fig. 1). For example, although not trivial, the identified relevant mechanisms can be investigated for their potential in robust industrial strains. Additionally, secondary effects of mutations that not only give a selective benefit, but also have a much broader impact, such as mutations in regulatory networks, can be prevented by only engineering the relevant trait. Strains constructed via ‘forward’ metabolic engineering can, after additional nontargeted modification of their genomes, be re-entered into the reverse metabolic engineering cycle. This can, for example, accelerate the evaluation of optimal gene sequences or pathway configurations (Fig. 1).

The unequivocal and fast identification of the genetic basis of improved performance remains the key challenge in reverse metabolic engineering of yeasts. In the 15 years since the first *S. cerevisiae* genome sequence was published, the toolbox for integral analysis of yeasts at different information levels (genome, proteome and metabolome) has rapidly expanded. In addition, the decreasing costs of several key analytical technologies are making them increasingly accessible for application in industrial and academic yeast research. This fast progress in tool development brings about a new challenge: How to make informed choices from a wide range of expensive analytical approaches? The goal of the present paper is not to exhaustively review the literature on reverse metabolic engineering of yeast. Instead, by discussing published examples on reverse metabolic engineering of yeast, we will identify advantages and limitations of the genome-wide analytical approaches that are currently available. Emphasis will be on analysis of yeast strains generated in ‘linear’ strain improvement programmes, for example, via classical mutagenesis or evolutionary engineering, rather than on the systematic exploration of yeast biodiversity.

## Genome expression analysis

Until recently, the costs of whole genome sequencing precluded its use as a routine laboratory technique in reverse metabolic engineering. Therefore, analysis of the molecular basis of industrially relevant traits has, for the past decade, strongly depended on genome expression studies. The goal of genome expression analysis in the context of reverse metabolic engineering is to correlate expression levels of individual genes with an industrially relevant performance parameter, such as productivity, yield, or robustness. These correlations form the basis for identification of lead genes and/or cellular processes, whose contribution to the phenotype of high-performing strains should subsequently be assessed by targeted genetic modification. As the coverage of state-of-the art proteomics platforms is still incomplete, transcriptome analysis is currently the only widely available means of truly genome-wide analysis of gene expression in yeast. We will therefore mainly focus our evaluation of genome expression for reverse metabolic engineering on transcriptome analysis. The potential added value of a few examples of proteomics and metabolomics in yeast reverse metabolic engineering is briefly discussed in separate paragraphs.

### Experimental design for genome expression analysis

DNA microarray analyses (DeRisi, 1997 Daran-Lapujade *et al*., 2009) and RNA sequencing (Wang *et al*., 2009b; Ozsolak & Milos, 2011) enable the rapid, quantitative, and inclusive correlation of the yeast transcriptome to environmental or genetic contexts. Furthermore, powerful algorithms enable analysis of the overrepresentation of functional categories (Kresnowati *et al*., 2006; Huang *et al*., 2009a, b) and transcription factor-binding sequences (Harbison *et al*., 2004; MacIsaac *et al*., 2006) that show a transcriptional up- or downregulation in a given context. Additionally, transcriptome data can be correlated with a vast body of information on the transcriptional responses of *S. cerevisiae* to a range of environmental parameters and genetic interventions. In the interpretation of transcriptome data, it is important to consider the context dependency of transcriptional regulation in yeast (Knijnenburg *et al*., 2008).

Cultivation conditions and specific growth rate have a substantial impact on yeast genome expression, which has been especially well documented for transcriptome analysis (Boer *et al*., 2003; Tai *et al*., 2005; Regenberg *et al*., 2006; Abbott *et al*., 2007; Castrillo *et al*., 2007; De Nicola *et al*., 2007; Fazio *et al*., 2008; Daran-Lapujade *et al*., 2009). This has important implications for the use of transcriptome data in reverse metabolic engineering. When experimental conditions and/or specific growth rates differ for the strains that are compared, this may generate nonproductive leads, that is, gene expression differences that do not reflect a positive contribution to the industrially relevant phenotype under study. Controlled cultivation in chemostat cultures avoids the impact of the changing environmental conditions that occur in batch cultivation, and the fixed dilution rate eliminates the impact of specific growth rate on transcriptome analyses of different yeast strains and environmental conditions (Daran-Lapujade *et al*., 2009).

An additional experimental design challenge, which is specifically associated with reverse metabolic engineering, arises when mutagenesis or laboratory evolution leads to ‘gain of function’ phenotypes. Ideally, transcriptome analysis should be performed under conditions where the trait of interest is expressed, but these do not always allow growth of the reference strain. For example, after laboratory evolution of strains for anaerobic growth on pentose sugars or for strongly induced ethanol or acetic acid tolerance (Sonderegger & Sauer, 2003; Stanley *et al*., 2010a; Wisselink *et al*., 2010; Wright *et al*., 2011), the reference strain cannot be grown under the conditions in which the selected phenotype becomes apparent. Consequently, the use of identical cultivation conditions that are permissive for both strains can only yield genes whose transcriptional up- or downregulation in the evolved strain does not depend on the conditions that led to its selection. An example of such ‘constitutive’ expression is the upregulation of transaldolase and transketolase-encoding genes in yeast strains selected for growth on xylose or arabinose (Wahlbom *et al*., 2003; Wisselink *et al*., 2010), which could already be observed in a comparison of glucose-grown cultures of the evolved and parental strains. Additional, important transcriptional changes may only be observable under conditions that are nonpermissive for the reference strain. For example, in an evolved l-arabinose-fermenting strain grown on glucose, transcript levels of the GAL regulon were the same as those in the nonevolved strain, presumably as a result of glucose repression (Wisselink *et al*., 2010). However, very high transcript levels of this regulon were observed during growth of the evolved strain on l-arabinose and, subsequently, linked to the deregulation of the *GAL2*-encoded transporter, which is responsible for l-arabinose transport in engineered *S. cerevisiae* strains (Becker & Boles, 2003; Wisselink *et al*., 2010). Similarly, *S. cerevisiae* strains whose acetic acid tolerance had been strongly increased by evolutionary engineering required induction by acetic acid to express the acquired hyper tolerance (Wright *et al*., 2011), thereby precluding a meaningful analysis of gene expression in cultures grown without acetic acid. In such cases, a three-way comparison can be applied by comparing genome expression of both the evolved and reference strains under conditions that are permissive for the reference strain with the evolved strain under the relevant condition (Wahlbom *et al*., 2003; van Maris *et al*., 2007; Wisselink *et al*., 2010). However, the inevitable consequence of such a comparison is that nonproductive leads are likely to be generated as a result of the different cultivation conditions.

### Interpretation of transcriptome data: sources of nonproductive leads

Studies in which analysis of genome-wide transcriptional responses were followed up by systematic analysis of the fitness of null mutants suggest that, generally, only a small fraction of transcriptionally responsive genes positively contribute to fitness under the conditions to which they showed a transcriptional response (Winzeler *et al*., 1999b; Birrell *et al*., 2002; Giaever *et al*., 2002, 2004; Tai *et al*., 2007). Also in transcriptome-based reverse metabolic engineering, the number of responsive genes usually far exceeds the number of productive leads, even when cultivation conditions are rigorously controlled and standardized. Several causes for nonproductive leads will be discussed below.

Nonproductive leads can occur when productive mutations directly or indirectly affect expression of other genes. A direct influence occurs, for example, when the productive mutation affects the *in vivo* activity of a transcriptional regulator that, in addition to genes that positively affect an industrially relevant phenotype, controls the expression of genes that do not. An example is provided by a study on a *S. cerevisiae* strain in which the sucrose-hydrolyzing enzyme invertase was relocated to the cytosol to improve ethanol yields on sucrose. Prolonged cultivation in sucrose-limited chemostat cultures led to a drastic improvement of the affinity for sucrose. Analysis of two independently evolved strains revealed increased transcript levels of many genes involved in maltose metabolism, whereas the improved affinity for sucrose could be entirely attributed to upregulation of a single maltose transporter gene (*MAL11*; Basso *et al*., 2011). Although the specific mutation responsible for the deregulation of MAL genes in the evolved strain was not identified, it seems plausible that it affected a transcriptional regulator. Similarly, the contribution of the very high expression of the entire GAL regulon in an *S. cerevisiae* strain evolved for fast l-arabinose fermentation (Wisselink *et al*., 2010) could be entirely explained from the essential role of Gal2p in l-arabinose transport (see above). The high expression levels of other GAL genes in the evolved l-arabinose-fermenting strain were most probably a side effect of a mutation in a regulatory protein whose primary evolutionary significance was the deregulation of *GAL2*. Although, in these cases, engineering of the regulator may reproduce the selected phenotype, identification and targeted engineering of the responsible reaction or transport step may be desirable, for example, to minimize protein burden (Snoep *et al*., 1995).

**Fig. 2:**
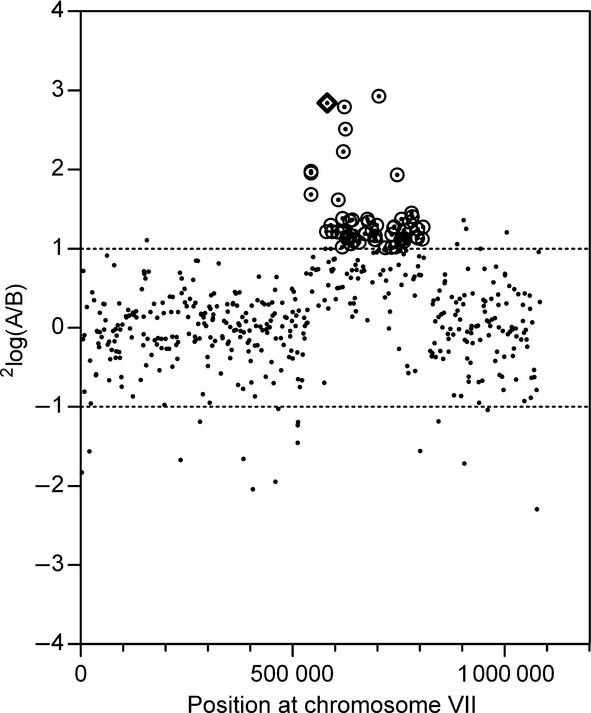
Transcriptome comparison of a genetically engineered *Saccharomyces cerevisiae* strain and an arabinose-fermenting derivative strain obtained by laboratory evolution. The dotted lines indicate a twofold difference. The circled dots represent genes located between positions 543 555 and 807 659 with an at least twofold increased transcript level in the evolved strain, indicating a duplication of a 250-kb region on chromosome VII. The increased expression level of YGR043C (diamond), encoding a transaldolase, was subsequently shown to contribute to enhanced arabinose fermentation rates in the evolved strain (reproduced with permission from Wisselink *et al*., 2010).

Another cause for nonproductive leads from transcriptome analysis is the deletion or amplification of multigene DNA fragments. Even in cases where the evolutionary distance between strains is small, as in laboratory evolution experiments with *S. cerevisiae*, which generally do not involve more than a few hundred generations of selective growth, deletion or amplification of multigene DNA fragments is frequently observed (Brown *et al*., 1998; Dunham *et al*., 2002; Jansen *et al*., 2005; Wisselink *et al*., 2010; Basso *et al*., 2011). In such cases, only a single gene on an amplified or deleted fragment may contribute to the phenotype of interest. By plotting transcript levels on a physical map of the yeast genome, amplified or deleted regions larger than a couple of genes stand out from the experimental background noise and can be identified directly. For example, in a transcriptome analysis of an *S. cerevisiae* strain evolved for fast anaerobic fermentation of l-arabinose, a 250-kb fragment of chromosome VII appeared to be duplicated (Wisselink *et al*., 2010; Fig. 2). One of the genes on this fragment (*YGR043C*) encodes a transaldolase isoenzyme and was subsequently shown to contribute to faster arabinose fermentation rates. Although the impact of other genes on the duplicated fragment was not studied, it seems plausible that their increased expression levels reflect ‘collateral damage’ of the duplication event that led to increased expression level of *YGR043C*.

Genetic differences that are related to the parameter of interest can sometimes be enriched by comparison of different independent strains that share the same industrially relevant phenotype. Of course, this does not provide leads if the evolved phenotype is caused by different mechanisms in the independent evolutions. A successful example of this approach is an early transcriptome-based reverse metabolic engineering study on strains with an evolved freeze resistance (Tanghe *et al*., 2002). A transcriptome comparison of three freeze-resistant strains with a freeze-sensitive reference strain enabled the demonstration that overexpression of a specific allele of the aquaporin-encoding *AQY2* gene led to increased freeze resistance in a naive strain. Recently, transcriptomes of three independently evolved *S. cerevisiae* strains with increased rates of galactose metabolism were compared with those of the nonevolved ancestor strain (Hong *et al*., 2011). Expression of genes controlled by the RAS/PKA pathway was found to be affected in all three evolved strains. When a specific point mutation in *RAS2* was introduced in the ancestor strain, this led to a significant increase in the specific growth rate on galactose (Hong *et al*., 2011).

In evolutionary engineering and in classical mutagenesis and selection, the relevant yeast strains generally share a common genetic background. Interpretation of transcriptome data becomes progressively more complicated when the genetic background of strains becomes more diverse. In addition to technical issues (e.g. the design of microarrays), the different ‘wiring’ of transcriptional regulation networks complicates interpretation of transcriptome data. However, even when different strain backgrounds are compared, excellent results are occasionally obtained when analysis is focused on subsets of genes that have been selected based on prior knowledge. A successful example is a transcriptome-based study on the molecular basis of tolerance to hydroxymethylfurfural (HMF), an important inhibitor of yeast fermentation in lignocellulosic hydrolysates (Petersson *et al*., 2006). On the basis of the knowledge that reduction of HMF to the corresponding alcohol is a key detoxification mechanism, a microarray-based transcriptome analysis of two nonrelated *S. cerevisiae* strains with different degrees of HMF tolerance focused on oxido-reductase-encoding genes. A set of 15 such genes were expressed at a higher level in the tolerant strain. Individual overexpression of these genes in an HMF-sensitive laboratory strain led to the identification of Adh6p as a major HMF reductase, whose overexpression led to increased rates of *in vivo* HMF reduction (Petersson *et al*., 2006). Similarly, a recent example is the identification of *ILV6* as a target to reduce diacetyl formation in lager brewers’ yeast through a combination of microarray-based comparative genome hybridization and a transcriptome analysis (Duong *et al*., 2011).

### The potential of proteome analysis in reverse metabolic engineering

A large and growing body of evidence shows that in *S. cerevisiae*, the correlation between transcript and protein levels is not perfect (de Groot *et al*., 2007; Wu *et al*., 2008; Rossignol *et al*., 2009; Olivares-Hernández *et al*., 2010; Rossouw *et al*., 2010), which indicates that, in many cases, regulation occurs at the level of translation and/or protein turnover. Consequently, analysis of gene expression at the level of transcription may overlook important changes, which might be detected by a thorough proteome analysis (Kolkman *et al*., 2005; Beck *et al*., 2011). However, we are aware of only very few studies in which proteome analysis has been performed in a reverse metabolic engineering context. A relevant study is a proteome analysis of a *S. cerevisiae* strain evolved for improved fermentation of xylose (Karhumaa *et al*., 2009). Because the same strain and its parental strain had previously been studied at the transcriptome level (Wahlbom *et al*., 2003), this investigation enabled a clear view on the additional information that can be gained from proteome analysis. Strikingly, the leads generated from the transcriptome and proteome comparisons showed very little correlation. Firstly, major increases were observed in the protein levels of the heterologous xylose reductase and xylitol dehydrogenase that were introduced into the ancestor strain via targeted genetic modification. No information on expression levels of their structural genes was obtained in the earlier transcriptome analysis, because they were not represented on the commercial *S. cerevisiae* microarrays. Secondly, six- to eightfold changes in the levels of several proteins – some of which were involved in key pathways of sugar metabolism – were not accompanied by significant changes in the corresponding mRNA (Karhumaa *et al*., 2009). This study reinforces the warning that, especially for central metabolic pathways, transcript levels cannot be considered as reliable indicators of either *in vivo* metabolic activity or protein levels (Daran-Lapujade *et al*., 2009). Clearly, proteomics analysis has high potential for use in reverse metabolic engineering, but requires further developments, such as increased coverage of the proteome and low-labor, high-throughput methodologies.

### Metabolite analysis to support lead generation

Quantitative measurements of intracellular metabolite levels can contribute to the identification of pathways whose capacity controls the rate of substrate consumption or product formation. Even though developments in (intracellular) metabolite analysis progress rapidly (Roessner & Bowne, 2009; Christen & Sauer, 2011), there are currently no methods available that enable the complete and accurate analysis of the yeast metabolome, and interpretation of intracellular metabolite data is complicated by the metabolic compartmentation of yeast cells. Moreover, metabolite analysis shares the challenges in experimental design that are inherent to all gene expression studies, such as context dependency or defining a reference situation for ‘gain of function’ phenotypes. Additionally, changes at the metabolite level alone are never sufficient to identify the underlying molecular mechanism. These limitations notwithstanding, in some studies metabolite analysis successfully resulted in the generation of leads for reverse metabolic engineering.

In genetically engineered strains of *S. cerevisiae* that were evolved for faster metabolism of xylose, analysis of intracellular metabolite levels indicated that the capacity of the nonoxidative pentose phosphate pathway is a key factor in engineering of pentose-fermenting strains (Zaldivar *et al*., 2002; Pitkänen *et al*., 2005). This indication is in line with metabolic engineering studies, which showed that overexpression of key enzymes of the pentose phosphate pathway is indeed essential to achieve high rates of pentose fermentation in *S. cerevisiae* (Hahn-Hägerdal *et al*., 2007; van Maris *et al*., 2007). However, as the metabolite studies cited above were not linked to transcriptome analysis or genome sequencing, improved performance of the evolved strains could not be linked to mutations or altered expression of specific genes.

A recent study on *S. cerevisiae* strains evolved for faster growth on galactose integrated different analytical approaches (Hong *et al*., 2011). Decreased intracellular concentrations of glucose-1-phosphate and galactose-1-phosphate, key metabolites of the Leloir pathway for galactose fermentation, coincided with an increased expression of *PGM2*, which encodes phosphoglucomutase. Identification of *PGM2* as a reverse engineering target was validated by previous work, which showed that its overexpression leads to increased rates of galactose metabolism in *S. cerevisiae* (Bro *et al*., 2005). In this case, metabolite analyses basically led to the confirmation of targets that would also have been identified by transcriptome analysis.

One of the major challenges in intracellular metabolite analysis is to assess whether altered metabolite concentrations are cause or consequence of an increased flux through a pathway. Integration of metabolite data with, for example, gene expression studies and a thermodynamic analysis can increase their predictive value for reverse metabolic engineering. For example, Wisselink *et al*. (2010) analyzed an engineered *S. cerevisiae* strain that was evolved for fast fermentation of l-arabinose via integrated analysis of the transcriptome and intracellular concentrations of intermediates of central carbon metabolism. A thermodynamic analysis based on measured intracellular metabolite concentrations of glycolysis and the pentose phosphate pathway indicated that the driving force for the transaldolase and transketolase reactions was much higher in arabinose-grown cultures of the evolved strain than in glucose-grown cultures of the evolved and parental strains. This suggested a limiting capacity of these two reactions. The two major genes for transaldolase and transketolase (*TAL1* and *TKL1*, respectively) were already strongly overexpressed in the evolved strain owing to previous targeted metabolic engineering (Wisselink *et al*., 2007). However, transcriptome analysis showed an increased expression in the evolved strain of two genes encoding ‘minor’ isoenzymes of transaldolase (*YGR043C*, Fig. 2) and transketolase (*TKL2*). Subsequent knockout studies confirmed the involvement of these genes in the improved arabinose fermentation kinetics of the evolved strain (Wisselink *et al*., 2010). Although these genes might also have been identified as targets for reverse engineering based on a transcriptome analysis only, their expression level was low relative to those of the ‘major’ *TAL1* and *TKL1* genes (Wisselink *et al*., 2010). In the examples discussed above, metabolite analysis led to improved understanding of the impact of various mutations on the biochemistry. Moreover, metabolite analysis provided additional, strong incentives to prioritize mutations for follow-up studies.

## Analysis of gene and genome sequences

In contrast to gene expression data, genome sequences of genetically homogeneous (‘pure’) cultures are context independent and offer a direct view on molecular changes at the DNA level. Furthermore, whereas a change in a single transcript, protein, or metabolite often has a drastic impact on the complete transcriptome, proteome, or metabolome, individual mutations will generally have little impact on the likelihood of mutations elsewhere on the genome. One notable exception to this are mutator phenotypes (Thompson *et al*., 2006; Raynes *et al*., 2011), in which a genetic change in one gene leads to an increased mutation frequency elsewhere on the genome and which may well be enriched for in classical strain improvement and evolutionary engineering.

Classical methods, such as genomic libraries and transposon mutagenesis, have been instrumental in identifying genotype–phenotype relations (Ross-Macdonald *et al*., 1999; de Jesus Ferreira *et al*., 2001; Jin *et al*., 2005; Ni *et al*., 2007; Hong *et al*., 2010). However, these techniques are often laboreous and can only identify dominant mutations. DNA sequencing is a powerful alternative, but the associated costs and the large size of the yeast genome in comparison with prokaryotes have long been prohibitive for its routine use in reverse metabolic engineering studies. Although the number of sequence- or hybridization-based reverse metabolic engineering studies with yeast is small, interesting insights into the potential of a sequence-based approach for the identification of reverse engineering targets that these studies provide is discussed below.

### (Re)sequencing of selected genes or plasmids

When available knowledge, models, flux analysis, or expression studies strongly point toward a certain gene or sequence, partial sequencing can sometimes economize the discovery of relevant mutations. This is exemplified by a recent study on the introduction of a heterologous phospho-*enol*-pyruvate carboxykinase (PCK) into *S. cerevisiae* as an alternative, ATP-efficient C3→C4 carboxylating pathway. Laboratory evolution was required to enable the heterologous PCK to functionally replace the *S. cerevisiae* pyruvate carboxylases. Based on a physiological analysis, it was hypothesized that the high activities of pyruvate kinase in *S. cerevisiae* might compete for phospho-*enol*-pyruvate with the heterologous PCK. Resequencing of the *PYK1* gene in the evolved strain revealed a point mutation, whose introduction in the nonevolved strain led to a reduced pyruvate kinase activity and enabled growth via the heterologous pathway (Zelle *et al*., 2010).

When heterologous enzymes or pathways are expressed from episomal vectors, it is straightforward to first establish, by plasmid curing and plasmid reintroduction into a naive strain, whether a mutation is chromosomal or plasmid borne. In the latter case, sequencing of the plasmid can be a fast, cost-effective alternative to whole genome analysis. In a recent study, a pyruvate carboxylase–negative mutant of *S. cerevisiae*, expressing an *Escherichia coli* malic enzyme gene, was evolved for growth on glucose as the sole carbon source. As in the previous example, the goal of this study was to explore energy-efficient pathways for production of C4-dicarboxylic acids (Zelle *et al*., 2011). After establishing that, in two independent mutants, the relevant mutations were plasmid borne, two different point mutations were identified in the *E. coli* gene. These were subsequently shown to drastically affect their redox cofactor preference, thereby enabling them to function in the carboxylating direction and to replace the yeast pyruvate carboxylase (Zelle *et al*., 2011). Scientific curiosity, time-to-results, and the disproportionality of the cost per base pair of sequencing specific genes or plasmids vs. whole genome analyses in the end determine the choice between these techniques.

### Hybridization-based microarray genome analysis

Comparative genome hybridization and oligonucleotide microarrays have proven to be a powerful tool in the discovery of variations between yeast strains (Winzeler *et al*., 1999a; Daran-Lapujade *et al*., 2003; Gresham *et al*., 2006; Schacherer *et al*., 2007, 2009), ranging from single-nucleotide polymorphisms to structural variations (Dunham *et al*., 2002; Gresham *et al*., 2010). DNA hybridization experiments assay the presence of complementary DNA that is present in a sample, usually on a DNA array. All array types, from BAC arrays to tiling arrays, can be used to detect structural variations between the sample and the reference on the chip. The density of DNA probes on the array determines the resolution of the analysis, with resequencing and tiling arrays having the ability to discover single-nucleotide variations (SNVs) (reviewed by Gresham *et al*., 2008a). Array-based genotyping has long had the advantage over whole genome resequencing that it is faster. Moreover, especially in dynamic experiments and in large comparative studies, the lower costs of array-based techniques provided an advantage (reviewed by Gresham *et al*., 2008a; but see next paragraph). However, these hybridization-based techniques have the inherent disadvantage that in quality as well as in quantity, they only allow a comparison with sequences that are represented on the microarray. Therefore, when used to analyze strains resulting from mutagenesis or evolutionary engineering, arrays need to be representative for the ancestor strains used in strain improvement. Additionally, while some types of microarray allow for the accurate mapping of the position of SNVs, determination of the exact identity of the sequence variation requires the use of either resequencing arrays or resequencing (see review Gresham *et al*., 2008a).

### Whole genome (re)sequencing of yeast strains for reverse metabolic engineering

The 1000-dollar genome (Mardis, 2006), an iconic target in human genomics, is rapidly becoming a reality for *S. cerevisiae*. Currently (September 2011), the costs for custom resequencing of a 12-Mb genome with short-read paired-end technology at 40+-fold coverage is about €850 (E. Zeinstra, B., Leiden, the Netherlands; personal communication). In principle, (re)sequencing of *S. cerevisiae* strains that have been obtained via evolutionary engineering or mutagenesis offers huge opportunities for reverse engineering.

The advantage of whole genome resequencing over array-based techniques with respect to the identification of SNVs was indicated by the discovery of additional mutations of yeast strains (Araya *et al*., 2010; Kvitek & Sherlock, 2011) that had previously been analyzed by tiling array-based genotyping (Gresham *et al*., 2008b; Kao & Sherlock, 2008). On the other hand, identification of structural variation with short next-generation sequence reads is challenging with current alignment techniques, but is likely to be solved with further technology improvements (reviewed by Alkan *et al*., 2011). For the reliable and comprehensive detection of relevant mutations, including structural rearrangements such as indels (insertions and deletions), inversions and duplications, it is not always sufficient to align and compare sequence data of a strain of interest to a reference genome, such as the first published *S. cerevisiae* genome (strain S288C, Goffeau *et al*., 1996).

To gain the full benefit of whole genome or whole transcriptome sequencing, it is important to have access to a well assembled and annotated genome sequence of the reference strain that is used as the ancestor in mutagenesis or evolutionary engineering experiments. Although new algorithms enable the *de novo* assembly of entire *S. cerevisiae* genomes from short-read sequence information only (see e.g. Nijkamp *et al*., 2010), reliable ‘gold standard’ genome sequences for reference strains will generally require sequencing of additional libraries. This will include, preferably, longer-read sequencing (classical Sanger sequencing; Roche 454 or new Pacific Biosystems) and mate-paired libraries of several insert sizes (ranging from 400 bp to 10 kbp) (Table 1) to close gaps in the assembled sequence and to obtain reliable assemblies of repetitive sequences (Argueso *et al*., 2009; Novo *et al*., 2009). The costs of fully assembling and annotating such a reference genome exceed the costs of routine resequencing, which limits the true availability of fully *de novo* assembled ‘gold standard’ genome sequences.

**Table 1:** Comparison of ‘next generation’ methods for whole genome sequence. Costs per megabase are estimated based on price quotes (September 2011) from companies that offer commercial sequencing services

Company (sequencing platform)	Chemistry	Read length (bp)	Total base count per run (Gb)	Accuracy (%)	Cost per Mb (€)
Illumina (Hiseq2000, GAIIX)	Reversible dye terminators	50–150	5	99.0	2.5
Life Technologies (ABI SOLiD, SOLiD4)	Oligonucleotide probe ligation	35–50	10	99.9	3
Roche (454 GS FLX Titanium)	Pyrosequencing	350–450	0.4	99.5	20
Pacific Biosystems (SMRT)	Phospho-linked fluorescent nucleotides	600–1400	100	85.0	15
Helicos Biosciences (HeliScope)	Reversible dye terminators	35	25	99.9	–

There are, hitherto, only few cases in which whole genome sequencing of *S. cerevisiae* has been performed in a reverse metabolic engineering context (Timmermann *et al*. 2010; Dhar *et al*., 2011; Hong *et al*., 2011; Kvitek & Sherlock, 2011). Timmermann *et al*. (2010) used whole genome sequencing to analyze the molecular basis for oxidative stress tolerance in a *S. cerevisiae* mutant that was obtained by mutagenesis with ethyl methanosulfonate. Comparison of raw sequence data, followed by a manual inspection of results for ambiguous sequence calls, yielded only four mutations that were predicted to cause amino acid changes in proteins. A mutation in the peroxiredoxin protein Tsa1 was subsequently shown to be responsible for the improved oxidative stress tolerance. The discovery of only four lead genes represents a marked contrast with many microarray-based transcriptome analyses, which typically yields dozens, if not hundreds of differentially expressed genes. This contrast was even clearer in a direct comparison of transcriptome analysis and whole genome sequencing in a study on *S. cerevisiae* strains evolved for faster growth on galactose (Hong *et al*., 2011). After selecting strains by 400 generations of growth on galactose as sole carbon source, hundreds of genes were differentially expressed relative to the parental strain. However, systematic analysis of whole genome sequencing data revealed only small numbers of nonconservative SNVs and insertions/deletions within genes (fewer than 20 in two of three strains). On the basis of the observation that mutations in genes involved in the RAS/PKA pathway occurred in all three strains, a specific point mutation in *RAS2* was reverse engineered in the ancestor strain and was shown to explain about half of the observed increase in growth rate on galactose (Hong *et al*., 2011).

**Fig. 3:**
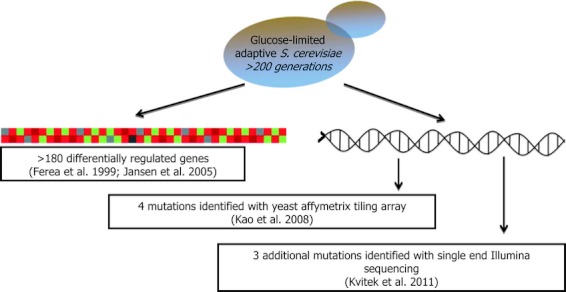
Analyses of evolved *Saccharomyces cerevisiae* strains adapted for glucose-limited cultivation conditions illustrate the advantages of whole genome sequencing over microarray-based transcriptome analysis in reverse metabolic engineering. Transcriptome analysis of four independently evolved strains consistently yielded more than 180 differentially expressed genes. Genotyping of a single cell line using tiling arrays and whole genome sequencing showed a much smaller number of underlying nonconservative mutations. Some mutations identified by whole genome sequencing went unnoticed by a previous analysis using tiling arrays.

Prolonged glucose-limited growth of *S. cerevisiae* in chemostat cultivation is a popular model for laboratory evolution and its molecular analysis. Transcriptome-based studies (Ferea *et al*., 1999; Jansen *et al*., 2005) identified hundreds of genes whose transcript levels changed as the yeast adapted to this nutrient limitation. In contrast, in a similar evolution experiment, resequencing of an adaptive clone revealed mutations in only six genes (Kvitek & Sherlock, 2011). Interestingly, a long terminal repeat insertion in *GPB2*, previously predicted based on tiling array analysis of this clone (Kao & Sherlock, 2008), was not identified during resequencing (Fig. 3). Mutations in *HXT6/7* and *GPB2* were shown to confer a statistically significant (*P* < 0.05) advantage over the ancestor strain during competitive glucose-limited cultivation (Kvitek & Sherlock, 2011). Unpublished results from our laboratory confirm that whole genome sequencing of parallel evolution lines can contribute to the rapid identification of key mutations in yeast strains generated in laboratory evolution experiments. For example, two suppressor mutants were isolated from independent laboratory evolution experiments with a *jen1* null mutant, which encodes the *S. cerevisiae* lactate transporter (Casal *et al*., 1999). These strains regained the ability to grow on lactate as sole carbon source through different point mutations in the same membrane transporter gene. Reverse engineering of these mutations confirmed that each of them enabled the transporter to act as an efficient lactate transporter. Interestingly, this gene was not identified as a target in a parallel transcriptome analysis (S. de Kok *et al*., in press).

## Discussion

An overview of the relevant literature enables two clear recommendations on experimental design of reverse metabolic engineering experiments of *S. cerevisiae*. Firstly, although the number of studies in which whole genome sequencing has been applied for the reverse metabolic engineering of yeasts is still small, the available information consistently indicates that this technique is a real game changer. In ‘linear’ strain improvement studies (e.g. chemical mutagenesis and laboratory evolution), whole genome sequencing typically yields many fewer lead genes than transcriptome analysis. Moreover, the changes that are identified at the DNA level can be immediately and exactly reconstructed in naive strain backgrounds. Also in view of its rapidly decreasing costs, whole genome sequencing should now be the first-choice analytical approach in reverse metabolic engineering of yeast strains. Metabolomics, proteomics, and transcriptomics can subsequently be used for further interpretation of genome sequencing data and to elucidate the biochemical impact of the mutation, but, in general, are less suitable as first-line analytical approaches than genome sequencing. An interesting development in this respect is the sequencing of mRNA (RNA-seq), because both genetic changes in coding sequences and the transcriptional responses are measured in one step (Wang *et al*., 2009b).

Secondly, prioritization of mutations is greatly facilitated by the use of parallel strain improvement experiments. Focusing on functional analysis of mutations that affect the same gene, pathway or cellular process in multiple independent evolution or mutagenesis experiments has been repeatedly shown to facilitate the fast identification of relevant targets (Zelle *et al*., 2010; Hong *et al*., 2011; S. de Kok *et al*., in press). Especially when the number of parallel strain improvement experiments is small, an exclusive focus on the ‘overlap’ of a small number of selected genotypes can potentially lead to loss of valuable information. Such a loss can result from mutations in different genes or processes that have a similar positive effect on the phenotype but which do not occur in all strains selected for analysis. Moreover, the phenotypic effect of a mutation, be it on evolutionary fitness or on industrial performance, can be strongly dependent on the genetic context. The relevance of this context dependency, which in genetics is known as epistasis, is illustrated by an elegant study by Kvitek & Sherlock (2011), who monitored the occurrence of mutations during laboratory evolution experiments with *S. cerevisiae* in glucose-limited chemostat cultures. The authors convincingly demonstrated that mutations in the hexose transporter gene *HXT6/7* and in the regulator gene *MTH1* exhibited negative epistasis: individual introduction of the mutations in a naive strain background led to an improved fitness, while their combined introduction had a negative effect. In addition to monitoring the incidence of mutations during evolution, increasing the number of parallel strain improvement experiments should facilitate identification of negatively epistatic mutations.

Wherever whole genome sequencing results in too many candidate leads, the power of molecular techniques should be amplified by their integration with classical yeast genetics. Analysis of segregation patterns after mating with a reference strain and systematic backcrossing can rapidly provide insight into the complexity of acquired genotypes and reduce the number of nonproductive mutations (Timmermann *et al*. 2010). Moreover, segregation of mutations in the offspring of a backcross with the strain of interest is a powerful technique in discovering positive epistasis (i.e. multigenic traits) through the identification of quantitative trait loci (Kearsey, 1998; Liti *et al*., 2009b; Ehrenreich *et al*., 2010; Cubillos *et al*., 2011; Parts *et al*., 2011). When knowledge on gene or protein function is limited, parallel strain improvement and identification of quantitative trait loci give information for target prioritization without requiring *a priori* knowledge on gene or protein function.

We anticipate that further technology developments and decreases in sequencing costs, combined with the automation of the parallel and combinatorial reconstruction of different genetic variations, will make reverse metabolic engineering one of the major driving forces in yeast biotechnology in the coming decade.
